# Severe diabetes and leptin resistance cause differential hepatic and renal transporter expression in mice

**DOI:** 10.1186/1476-5926-11-1

**Published:** 2012-04-23

**Authors:** Vijay R More, Xia Wen, Paul E Thomas, Lauren M Aleksunes, Angela L Slitt

**Affiliations:** 1 Department of Biomedical and Pharmaceutical Sciences, University of Rhode Island, Kingston, RI, 02881, USA; 2 Department of Pharmacology and Toxicology, Rutgers University Ernest Mario School of Pharmacy and Environmental and Occupational Health Sciences Institute, Piscataway, NJ, 08854, USA; 3 Department of Chemical Biology, Rutgers University Ernest Mario School of Pharmacy and Environmental and Occupational Health Sciences Institute, Piscataway, NJ, 08854, USA

**Keywords:** Leptin, Diabetes, Transporters, Disposition, Acetaminophen

## Abstract

**Background:**

Type-2 Diabetes is a major health concern in the United States and other Westernized countries, with prevalence increasing yearly. There is a need to better model and predict adverse drug reactions, drug-induced liver injury, and drug efficacy in this population. Because transporters significantly contribute to drug clearance and disposition, it is highly significant to determine whether a severe diabetes phenotype alters drug transporter expression, and whether diabetic mouse models have altered disposition of acetaminophen (APAP) metabolites.

**Results:**

Transporter mRNA and protein expression were quantified in livers and kidneys of adult C57BKS and db/db mice, which have a severe diabetes phenotype due to a lack of a functional leptin receptor. The urinary excretion of acetaminophen-glucuronide, a substrate for multidrug resistance-associated proteins transporters was also determined. The mRNA expression of major uptake transporters, such as organic anion transporting polypeptide Slco1a1 in liver and kidney, 1a4 in liver, and Slc22a7 in kidney was decreased in db/db mice. In contrast, Abcc3 and 4 mRNA and protein expression was more than 2 fold higher in db/db male mouse livers as compared to C57BKS controls. Urine levels of APAP-glucuronide, -sulfate, and N-acetyl cysteine metabolites were higher in db/db mice.

**Conclusion:**

A severe diabetes phenotype/presentation significantly altered drug transporter expression in liver and kidney, which corresponded with urinary APAP metabolite levels.

## Background

The prevalence of obesity and metabolic syndrome has increased at an alarming rate. By the year 2030, the number of adults with either type-1 or type-2 diabetes is estimated to be greater than 350 million [1]. Adult onset type-2 diabetes (T2DM) constitutes over 90% of all diabetes cases and is characterized by insulin resistance, abnormal insulin secretion, or both. Of these cases, it is estimated that 16% of people have undiagnosed or poorly managed diabetes (NIDDK National Health Interview survey, 2007–2009).

It is well documented that Type-2 diabetes and hepatic steatosis are co-present [2]. The incidence of non-alcoholic fatty liver disease (NAFLD) is prevalent in 40 to 70% of patients with T2DM [3,4]. This type of liver disease originates as hepatic steatosis, and can progress to non-alcoholic steatohepatitis (NASH), cirrhosis, and end stage liver failure [5]. T2DM-related NAFLD is not fully understood, but it is known that leptin and insulin are important mediators in the progression of NAFLD [6]. Leptin is a hormone secreted by adipocytes, which binds to the leptin receptor and increases partitioning of fatty acids towards oxidation instead of triacylglycerol formation [7]. In mice and rats, leptin deficiency causes hyperphagia and obesity [8]. Moreover, the lack of leptin action causes increased insulin secretion, which is hypothesized to cause insulin resistance in rodents and humans [9]. Insulin resistance syndrome is hypothesized to cause NAFLD and augment progression to NASH [10].

T2DM and hepatic steatosis are modeled by a variety of diet and genetically modified rodent models. Db/db mice (BKS.Cg-m +/+ Leprdb/J) mice possess a spontaneous diabetes (Db) mutation in the leptin receptor. Db/db mice are insulin resistant, hyperinsulinemic, hyperglycemic, glucose intolerant, and possess abnormal islet cell morphology [11-13]. They become hyperinsulinemic from 10–14 days after birth; and exhibit significant weight gain with abnormally high triglycerides and low- and very low-density lipoproteins at 3 to 4 weeks of age. Hyperglycemia appears after 4–6 weeks of age.

Other mouse models of obesity, diabetes, and NAFLD exhibit altered transporter expression in liver and kidney [14]. Transporters are membrane proteins, which facilitate chemical transport into and out of cells [15]. Organic anion transporting polypeptides, organic anion transporters and organic cation transporters are often referred to as “uptake transporters”. They are predominantly localized to the basolateral membrane and extract chemicals from blood into hepatocytes (as reviewed by [15,16], but it should be noted that Oatps are localized to the luminal membrane in kidney [17]. Transporters that are members of the ATP-binding cassette (Abc) superfamily facilitate efflux of chemicals out of cells; and include Multidrug resistance proteins (Abcbs), Multidrug resistance-associated proteins (Abcc), Bile salt-export pump (Abcb11), and Breast cancer resistance protein (Abcg2). In liver, Abcc2, Abcg2 and Abcbs are localized to the canalicular membrane and facilitate biliary excretion of chemicals. Abcc1, 3–6 are localized sinusoidally and/or basolaterally, and efflux chemicals from hepatocytes into blood. In kidney, organic anion and cation transporters contribute to renal clearance, along with organic anion transporting polypeptides and Abcc transporters for determining the urinary excretion of many endogenous chemicals and xenobiotics.

There is evidence in rodents and humans that obesity, NAFLD, and NASH may increase susceptibility to drug-induced liver disease (DILI) [18] and exhibit altered excretion of acetaminophen [19]. Early studies demonstrated that obese overfed rats, which display NAFLD, were more sensitive to acetaminophen (APAP)-induced liver toxicity [18]. Other studies have demonstrated that obese rats exhibited increased furosemide-induced renal and hepatic toxicity [20], as well as gentamicin-induced nephrotoxicity [21]. More recently, studies documented higher serum and urinary levels of APAP glucuronide (APAP-G) in children with NAFLD, as compared to controls, after a single dose of APAP [22].

Because obese and diabetic people comprise a significant portion of the population within the United States, there is a growing need to better predict drug clearance, DILI, adverse drug effects, and drug efficacy in this population. As transporters comprise a significant mechanism by which multiple drugs undergo hepatic and renal clearance, it is imperative to determine whether diabetes affects transporter expression. The purpose of this study was to compare drug transporter expression levels in normal and diabetic mice and illustrate that the disposition of a prototypical Abcc substrate is altered. The study herein thoroughly characterizes drug transporter expression in the db/db model, which can provide guidance for disposition/toxicology studies in diabetics. In the present study, transporter mRNA and protein expression was markedly changed in db/db mice, which exhibit a severe diabetes phenotype and NAFLD. Moreover increased excretion of APAP metabolites into urine was observed in db/db mice.

## Results

### Tissue and body weights, blood glucose levels, and liver histopathologic evaluation in C57BKS and db/db mice

Table 1 illustrates the body weights, liver and kidney weights and blood glucose levels of C57BKS and db/db mice at 9 weeks of age. Body weights for db/db mice were 1.7 and 2.1 times higher than C57BKS males and females, respectively. Db/db male and female mice had liver weights that were 1.8 and 2.1 times greater than those of C57BKS mice, respectively. At nine weeks of age, blood glucose levels in db/db mice were elevated about 3-fold.

**Table 1:** **Body, liver and kidney weight and blood glucose levels for db/db and C57BKS control mice**^**a**^

**Strain**	**Gender**	**Liver Weight (g)**	**Kidney weight (g)**	**Average body weight (g)**	**Liver/Body weight**	**Mean blood glucose levels (mg/dL)**
C57BKS	Female	0.89 ± 0.03	0.25 ± 0.00	17.75 ± 0.23	0.050 ± 0.001	156 ± 3
	Male	1.00 ± 0.02	0.31 ± 0.02	21.89 ± 0.35	0.046 ± 0.001	158 ± 9
Db/db	Female	1.88 ± 0.08*	0.28 ± 0.01	37.71 ± 0.60*	0.050 ± 0.001	442 ± 48*
	Male	1.87 ± 0.06*	0.33 ± 0.01	38.67 ± 0.44*	0.048 ± 0.001	455 ± 33*

^a^Livers, kidneys, and blood were collected from C57BKS and db/db mice at 9 weeks of age. (*) indicates values significantly different from control (p ≤ 0.05). All weights expressed in grams ± SEM. Asterisk (*) represents statistically significant difference of parameters between C57BKS and db/db mice (p≤0.05).

Histopathological analysis showed mild to moderate steatosis in male and female db/db mice (Additional file 1: Figure S1). Both male and female db/db mice exhibited centrilobular and midzonal hepatocyte microvesicular vacuolation. Livers of C57BKS mice appeared normal without vacuolations.

### Db/db mice exhibit altered uptake transporter mRNA and protein expression in liver

**Figure 1:**
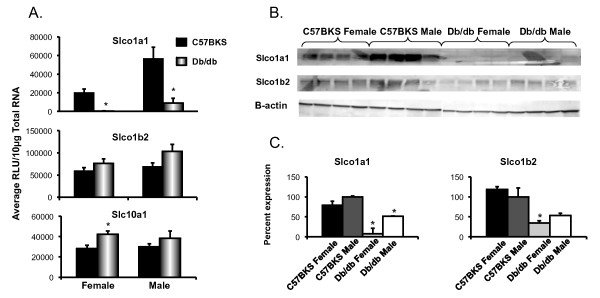
**Uptake transporters Slco1a1, 1b2 and Slc10a1 expression in livers of C57BKS and db/db mice (n = 8). A**) Messenger RNA expression for Slco1a1, 1b2 and Slc10a1. Total RNA was isolated from livers of adult db/db and C57BKS mice, and mRNA was quantified using the Branched DNA signal amplification assay. The data is plotted as average Relative Light Unit (RLU) per 10μg total RNA ± SEM. Asterisks (*) represent a statistically significant expression difference between db/db mice and C57BKS control mice of same gender (p≤0.05). Number signs (#) represent a significant expression difference between genders, i.e. male and female C57BKS or male and female db/db mice. **B**) Slco protein identification and quantification by western blot in crude membrane fractions from livers of C57BKS and db/db mice. Proteins (75 μg/lane) were separated on 4–20% acrylamide/bis PAGE, transblotted, incubated with primary and secondary antibodies, and visualized by fluorescence. **C**) Quantification of western blots by using the Quantity One® software (Biorad, Hercules, CA). The average band intensity for C57BKS males was set to 100% and other groups were normalized to that density. Asterisks (*) represent a statistically significant difference between average band intensity as compared to that of C57BKS males (p≤0.05). Slco1a1 mRNA and protein expression were downregulated in both male and female db/db mice as compared to controls. Slco1a4 (data not shown) and 1b2 mRNA expression remained unchanged but Slco1b2 protein expression was downregulated in db/db females. Slc10a1 mRNA expression was upregulated in db/db females as compared to C57BKS females.

Solute carrier proteins are predominantly localized to the basolateral membrane of hepatocytes and transport chemicals into the hepatocytes and are generally referred to as uptake transporters. Slco1a1 expression was higher in male C57BKS mice than in female C57BKS mice (Figure 1A), which is consistent with C57Bl/6 mice [23]. Slco1a1 mRNA expression was markedly downregulated in livers of male and female db/db mice. Slc10a1 (Ntcp) mRNA expression was increased in db/db females as compared to C57BKS females.

Figure 1B illustrates the relative protein expression of Slco1a1 and 1b2 in crude membrane fractions isolated from livers of C57BKS and db/db mice. Figure 1C shows the quantification of western blots in Figure 1B. Slco1a1 protein levels were markedly downregulated in livers of db/db mice. Slco1b2 protein expression in liver was also markedly downregulated by about 50% in db/db males and females as compared to C57BKS mice.

### Db/db mice exhibit altered efflux transporter mRNA and protein expression in liver

**Figure 2:**
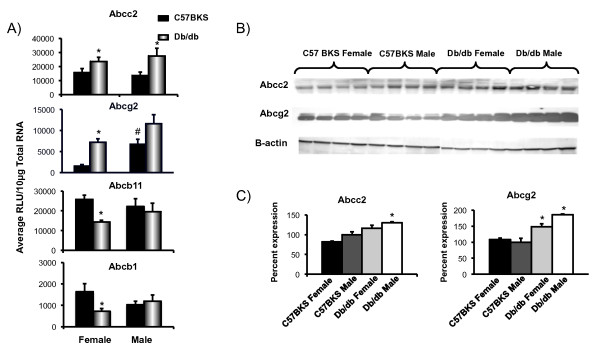
**Canalicular efflux expression in liver of db/db and C57BKS mice. A**) Messenger RNA expression for Abcc2, Abcg2, Abcb11 and Abcb1. Total RNA was isolated from liver, and mRNA was quantified using branched DNA signal amplification assay. The data plotted as average Relative Light Unit (RLU) per 10 μg total RNA ± SEM. Asterisks (*) represent a statistically significant expression difference between C57BKS and db/db mice of same gender (p≤0.05). Number signs (#) represent a statistically significant expression gender difference between male and female db/db mice, or male and female C57BKS mice. **B**) Abcc2 and Abcg2 protein identification and quantification by western blot in crude membrane fractions from livers of C57BKS and db/db mice. Proteins (75μg/lane) were separated on 4–20% acrylamide/PAGE, transblotted, incubated with primary and secondary antibodies, and visualized by fluorescence. **C**) Quantification of western blots by using the Quantity One® software (Biorad, Hercules, CA). The average band intensity for C57BKS males was considered 100% and other groups were compared with that density. Asterisks (*) represent a statistically significant difference between average band intensity as compared to that of C57BKS males (p≤0.05). Abcc2 mRNA expression increased in both male and female db/db mice, whereas protein expression increased in db/db males as compared to respective controls. In db/db females, both mRNA and protein expression of Abcg2 was upregulated, and in db/db males, Abcg2 mRNA was not significantly upregulated but the protein was significantly up. Abcb11 and Abcb1 mRNA expression was down in db/db females as compared to C57BKS females.

Multidrug resistance-associated proteins are efflux transporters that facilitate efflux of chemicals out of hepatocytes into bile or blood. Figure 2 illustrates mRNA and protein expression of Abc transporters localized to the canalicular membrane in livers of db/db and C57BKS mice. Abcg2 mRNA expression was higher in C57BKS males than C57BKS females. Abcc2 mRNA levels in livers of db/db males and females were 2 and 1.5 fold higher than C57BKS males, respectively. Abcc2 protein expression was also upregulated in db/db males as compared to C57BKS mice. Abcg2 mRNA and protein expression also increased with the diabetes phenotype, wherein mRNA expression doubled in db/db males and females. Correspondingly, Abcg2 protein levels were increased by 50% and 100% in livers of db/db male and female mice, respectively. Abcb11 and Abcb1 mRNA expression was decreased in db/db females as compared to C57BKS females.

**Figure 3:**
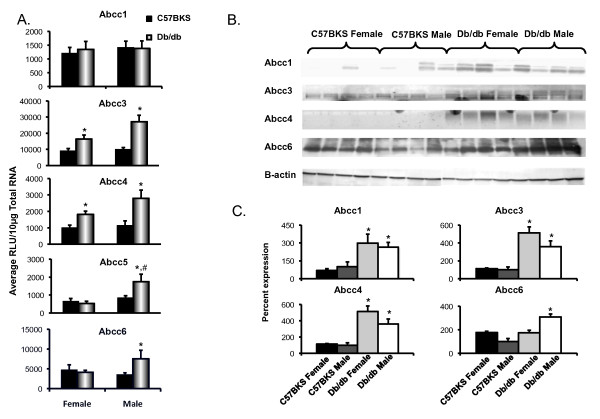
**Multidrug resistance-associated protein Abcc1, 3–6 expression in livers of C57BKS and db/db mice. A**) Messenger RNA expression for Abcc1, 3, 4, 5 and 6. Total RNA was isolated from livers of adult db/db and C57BKS mice, and mRNA was quantified using branched DNA signal amplification assay. The data plotted as average Relative Light Unit (RLU) per 10 μg total RNA ± SEM. Asterisks (*) represent a statistically significant difference of expression between C57BKS and db/db mice of same gender (p≤0.05). Number sign (#) represents statistically significant expression difference between male and female db/db mice and male and female C57BKS mice. **B**) Abcc1, 3, 4, and 6 identification and quantification by western blot in crude membrane fractions from livers of C57BKS and db/db mice. Proteins (75 μg/lane) were separated on 4–20% acrylamide/bis PAGE, transblotted, incubated with primary and secondary antibodies and visualized by fluorescence. **C**) Quantification of western blots by using the Quantity One® software (Biorad, Hercules, CA). The average band intensity for C57BKS males was considered 100% and other groups were compared with that density. Asterisks (*) represent a statistically significant difference between average band intensity as compared to that of C57BKS males (p≤0.05). Abcc1 mRNA was unchanged but protein expression was upregulated in both male and female db/db mice. Abcc3 and 4 mRNA as well as protein expression was upregulated in both male and female db/db mice. Abcc5 and 6 mRNA expression was upregulated in db/db males, but remained unchanged in females.

Figure 3A illustrates mRNA expression for efflux transporters localized to the sinusoidal and/or basolateral membrane. Db/db males have higher expression of Abcc5 than db/db females. In general, db/db mice display increased Abcc transporter expression as compared to C57BKS mice. Db/db male mice expressed Abcc3 and 4 mRNA levels in liver that were 2.7 and 2.4 fold higher, respectively, than C57BKS males. Db/db female mice expressed Abcc3 and 4 mRNA almost 1.8 fold more than C57BKS females. Abcc5 mRNA expression in liver was unchanged in females, but was increased 1.3-fold in livers of db/db males. Abcc6 mRNA expression was unaltered in livers of db/db females, but was 2.1 fold higher in db/db males than that in C57BKS males.

**Figure 4:**
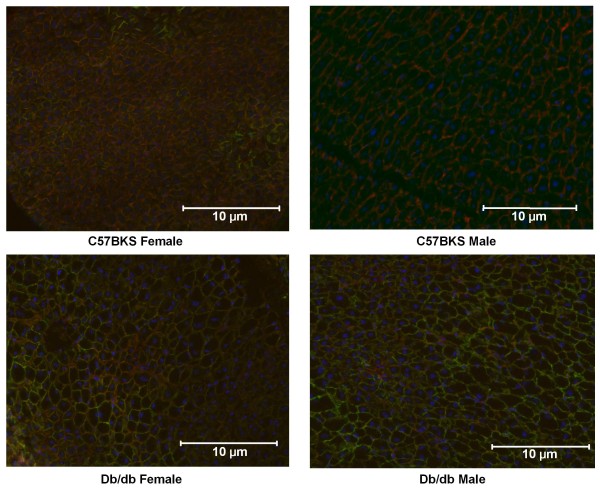
**Immunohistochemical staining of liver sections for Abcc3 detection.** Frozen livers were cut to 5 μm cryosections and fixed in 4% paraformaldehyde in phosphate-buffered saline (PBS). Sections were blocked in goat serum followed by incubation with *anti*-Abcc3. Sections were washed with PBS and incubated with goat *anti*-rat IgG conjugated to Alexfluor 488 (green staining) and rhodamine-conjugated phalloidin (red). Sections were then rinsed with PBS/T, PBS, and water, air dried, and then mounted with Prolong Gold containing DAPI (blue staining). All images are displayed as 200X magnification. It was observed that green staining displaying Abcc3 expression was higher in db/db male and female mice as compared to controls.

Figures 3B and C illustrate Abcc protein expression in crude membrane fractions isolated from livers of male and female db/db and C57BKS mice. Abcc1, 2, 4 protein expression in liver did not differ between males and females. Abcc6 protein expression in liver was higher in females than males. Abcc1 protein expression was significantly upregulated by 5- and 2.6-fold in male and female db/db mice, respectively. Liver Abcc3 and 4 protein expression was 3–4 fold higher in db/db mice compared to C57BKS mice. Increased sinusoidal/basolateral Abcc3 staining was also observed in livers of male db/db mice (Figure 4). The staining observed was consistent with that previously reported [24]. Db/db females also expressed increased Abcc6 protein levels in liver did not differ between db/db and C57BKS mice.

### Db/db mice exhibit altered transporter mRNA and protein expression in kidney

**Figure 5:**
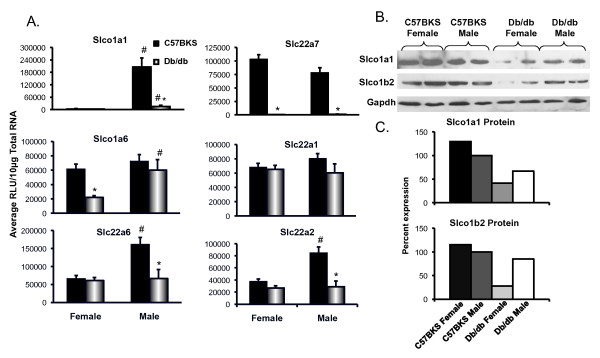
**Uptake transporter Slco1a1, 1b2, 1a6, Slc22a6, Slc22a7, Slc22a1 and Slc22a2 expression in kidneys of C57BKS and db/db mice. A**) Messenger RNA expression of uptake transporters in kidneys of C57BKS and db/dB mice. Total RNA was isolated from kidneys of these mice, and mRNA was quantified by the branched DNA signal amplification assay. The data is plotted as average RLU per 10 μg total RNA ± SEM. **B**) Protein expression of Slco1a1 and 1b2 in crude membrane fractions from kidneys of C57BKS and db/db mice (n = 2). Proteins (75 μg/lane) were separated on 4–20% acrylamide/bis PAGE, transblotted, incubated with primary and secondary antibodies and visualized by fluorescence. **C**) Quantification of western blots by using the Quantity One® software (Biorad, Hercules, CA). The average band intensity for C57BKS males was considered 100% and other groups were compared with that density. Asterisks (*) represent a statistically significant expression difference between db/db mice and C57BKS control mice of the same gender (p≤0.05). Number signs (#) represent a statistically significant expression difference between male and female db/db mice or male and female C57BKS mice (p≤0.05). Slc22a7 mRNA expression was downregulated in db/db male and female mice. Slco1a1, Slc22a2 and 22a6 mRNA expression was downregulated in db/db males as compared to C57BKS males. Slco1a1, Slc22a2 and 22a6 mRNA expression was more in C57BKS males as compared to C57BKS females. Slco1a1 and 1b2 protein expressions were significantly decreased in db/db females as compared to C57BKS females.

**Figure 6:**
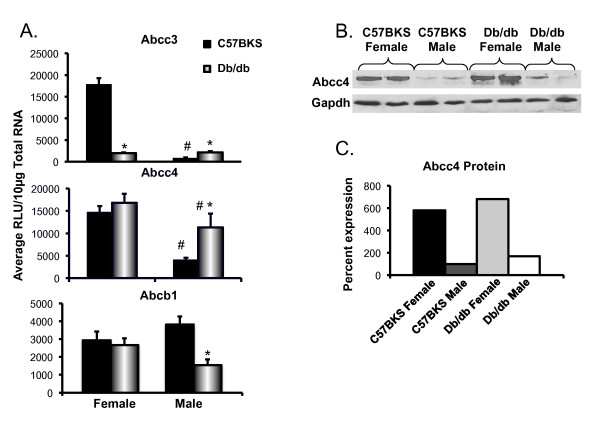
**Efflux transporter expression in kidneys of C57BKS and db/db mice. A**) Messenger RNA expression of Abcc3, 4 and Abcb1. Total RNA was isolated from kidneys of adult db/db and C57BKS mice, and mRNA expression was quantified using the branched DNA signal amplification assay. The data plotted as average RLU per 10 μg total RNA ± SEM. **B**) Protein expression of Abcc4 from crude membrane fractions of kidneys of C57BKS and db/db mice (n = 2). Proteins (75 μg/lane) were separated on 4–20% acrylamide/bis PAGE, transblotted, incubated with primary and secondary antibodies and visualized by fluorescence. **C**) Quantification of western blot by using the Quantity One® software (Biorad, Hercules, CA). The average band intensity for C57BKS males was considered 100% and other groups were compared with that density. Asterisks (*) represent a statistically significant expression difference between db/db mice and C57BKS mice of the same gender (p≤0.05). Number signs (#) represent a statistically significant expression difference between male and female db/db mice or male and female C57BKS mice. Abcc3 expression was downregulated in db/db females and upregulated in db/db males as compared to respective controls. Abcc4 mRNA expression was upregulated in db/db males as compared to C57BKS males. Abcc1, 2, Abcg2 mRNA expression also remained unchanged in kidneys of these mice (data not shown).

Slco1a1, 1a6, Slc22a1, Slc22a2, Slc22a6, Slc22a7, Abcc1-4, Abcb1, Abcg2 mRNA expression was quantified in kidneys of db/db and C57BKS mice (Figures 5 and 6). Basal expression of Slco1a1 mRNA in males was more than females, in both phenotypes. Also, Slc22a2 and 22a6 mRNA was expressed more in C57BKS males than C57BKS females. Slco1a1 mRNA expression was significantly lower in kidneys of db/db than that expressed in C57BKS mice, with expression approaching undetectable levels. Slco1a1 protein expression was also decreased in db/db females as compared to C57BKS females. In female db/db mice, Slco1a6 mRNA expression was decreased to only about 40% of that detected in kidneys of C57BKS females. Slc22a7 expression was markedly lower in kidneys of male and female db/db mice as compared to C57BKS controls. Slc22a6 mRNA expression was unchanged in kidneys of db/db females, but in db/db males was significantly reduced to about one third of that expressed in kidneys of male C57BKS mice. Slc22a2 mRNA expression was decreased to about 25% of controls in kidneys of male db/db mice, but was similarly expressed in kidneys of db/db and C57BKS females. Slc22a1 mRNA expression in kidneys was similar between genotypes.

Among efflux transporters, expression of Abccs was altered in kidneys of db/db mice. Db/db females exhibited marked down regulation of Abcc3 mRNA in kidney compared to C57BKS female mice. Basal Abcc3 mRNA expression was markedly higher in female kidneys as compared to male kidneys, which is consistent with other published studies [14]. Abcc4 mRNA expression was unchanged in db/db females, but was significantly increased, by almost 3-fold in kidneys of db/db male mice, as compared to that detected in male C57BKS mice. Also, basal expression of Abcc4 mRNA as well as protein in female kidney was almost 3-fold higher than that expressed male kidney. Abcc2 mRNA expression in kidney did not differ between db/db and C57BKS mice for either gender (data not shown).

### Db/db mice exhibit altered nuclear receptor and receptor target gene expression

**Figure 7:**
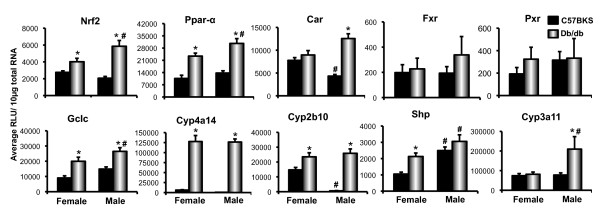
**Trascription factor Nrf2, and nuclear receptor Ppar-α, Fxr, Pxr, Car and their target genes mRNA expression in livers of C57BKS and db/db mice.** Messenger RNA expression of Nrf2, Ppar-α, Fxr, Pxr, Car, Gclc, Cyp4a14, Cyp2b10, Cyp3a11 and Shp was quantified. Total RNA was isolated from livers of adult db/db and C57BKS mice, and mRNA expression was quantified using the branched DNA signal amplification assay. The data plotted as average RLU per 10 μg total RNA ± SEM. Asterisks (*) represent a statistically significant expression difference between db/db mice and C57BKS mice of the same gender (p≤0.05). Number signs (#) represent a statistically significant expression difference between male and female db/db mice or male and female C57BKS mice. Nrf2 and its target gene Gclc display increase in male as well as female db/db mice, as compared to respective C57BKS controls. Similarly, Ppar-α and Cyp4a14 expression also increased in db/db mice. Car expression was increased in male db/db mice, and its target gene Cyp2b10 expression was also increased in male as well as female db/db mice. Fxr expression was unaltered in both male and female db/db mice, however, Shp, a target gene of Fxr, displayed increase in female db/db mice as compared to C57BKS females. Similarly, Pxr expression wasn’t altered, however, it’s target gene Cyp3a11 expression was increased in male db/db mice.

The relative expression of the transcription factor, nuclear factor E2 related factor 2 (Nrf2), as well as nuclear hormone receptors peroxisome proliferator activated receptor alpha (Ppar-α), constitutive androstane receptor (Car), farnesoid-X-receptor (Fxr) and pregnane-X-receptor (Pxr) mRNA expression was quantified in livers of db/db mice (Figure 7). In both male and female db/db mice, Nrf2 mRNA expression was significantly increased compared to C57BKS controls. Glutamate cysteine ligase (Gclc), a Nrf2 target gene, was correspondingly increased in livers of db/db mice. Ppar-α, and its target gene Cyp4a14 mRNA expression were also higher in male and female db/db mice as compared to C57BKS mice. Similarly, Car and Cyp2b10 expression also increased in male db/db mice as compared to C57BKS. Female db/db mice also displayed increased Cyp2b10, however, Car was unchanged. Pxr mRNA expression was not altered, however, its target Cyp3a11 expression was increased in db/db males. Similarly, Fxr mRNA did not increase significantly, however, one of its target genes, small heterodimer partner (Shp) was increased in db/db females compared to C57BKS females.

### Db/db mice exhibit increased urine APAP and APAP metabolites levels, and enhanced expression of UDP glucuronosyl transferase (Ugt) 1a6 and sulfotransferase (Sult) 1a1

**Figure 8:**
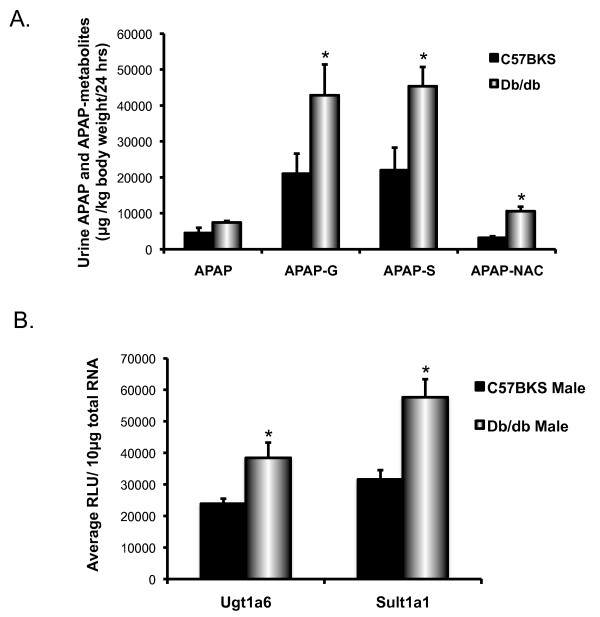
**Urine acetaminophen (APAP) and acetaminophen metabolite concentrations and APAP metabolizing enzymes expression in male C57BKS and db/db mice. A**) Urinary levels of APAP and its conjugation metabolites glucuronide, sulfate, and N-acetyl cysteine levels in male C57BKS and db/db mice. Acetaminophen (150 mg/kg, po) was administered to C57BKS and db/db male mice (n = 5), mice were housed in metabolic cages and urine was collected for 24 hrs. Urine proteins were precipitated by methanol precipitation and the extracted samples analyzed by HPLC. Asterisks (*) represent a statistically significant concentration difference between C57BKS and db/db mice (p≤0.05). APAP-glucuronide (APAP-G), sulfate (APAP-S), and N-acetyl L-cysteine were detected in higher amounts in urine of db/dB mice as compared to C57BKS. **B**) Messenger RNA expression of Ugt1a6 and Sult1a1 in livers of male C57BKS and db/db mice. Total RNA was isolated from livers of adult db/db and C57BKS male mice, and mRNA expression was quantified using the branched DNA signal amplification assay. The data plotted as average RLU per 10 μg total RNA ± SEM. Asterisks (*) represent a statistically significant expression difference between db/db mice and C57BKS mice of the same gender (p≤0.05). Both Ugt1a6 and Sult1a1 mRNA expression was increased significantly in livers of male db/db mice as compared to C57BKS mice.

Prior work in male rats demonstrated that APAP-G is a substrate for mouse and rat Abcc3 [25], and induction of Abcc3 expression in liver is associated with increased vectorial excretion of APAP-G [26,27]. Additionally, in mice, Abcc3 and 4 contribute to the basolateral excretion of APAP-sulfate (APAP-S) [25]. Because of Abcc3 and 4 transporters expression was significantly elevated in livers of db/db mice, and Abcc4 expression was significantly elevated in kidney, an additional study aimed to explore whether APAP-G and –S excretion into urine was increased. Therefore, a low, non-toxic APAP dose (100 mg/kg, po) was administered to male C57BKS and db/db mice, and of the total amount of urine APAP-G and APAP-S was quantified 24 hours after administration (Figure 8A). Urine flow rates were average 1 mL/24 hr for C57BKS and 2.7 mL/24 hr for db/db male mice. Taking differences in body weight into account, urine APAP-G and APAP-S amounts in urine were twice as high as that in urines from C57BKS mice. Thus, cumulative excretion of APAP conjugation metabolites was higher in db/db mice. As Ugt1a6 and Sult1a1 are primary conjugation enzymes for APAP-G and APAP-S production [28,29], their mRNA expression was evaluated (Figure 8B). Ugt1a6 and Sult1a1 mRNA expression was increased in male db/db mice as compared to C57BKS mice, which corresponded with increased APAP-G and APAP-S levels in urine.

## Discussion

The current study demonstrates that db/db mice, which are a widely used rodent model of diabetes with excessive weight gain and NAFLD, display profound alteration of transporter expression in both liver and kidney at the level of mRNA and protein expression. These observations are in agreement with [14] and [30]. Increased urine APAP-G and –S levels were also observed, which consistent with enhanced APAP-G disposition observed in other rodent steatosis models [19]. Slco1a1 expression was markedly downregulated in livers and kidneys of db/db mice. As Slco1a1 mediates transport of wide variety of anionic, cationic, zwitterionic, as well as, neutral chemicals [31], a significant decrease in Slco1a1 expression in liver and kidney could cause marked changes in pharmacokinetics and toxicity in the db/db mouse model. Along with Slco1a1, Slco1b2 protein expression was significantly decreased in livers of db/db female mice. In mice, Slco1a1, transports similar substrates as SLCO1A2, 1B1 and 1B3 in humans [32]. As Ppar-α has a central role in the down regulation of Slco1a1 in mouse liver [33,34], and is upregulated in db/db liver, according to present study as well as previous findings [35], it is possible that the observed downregulation is via a Ppar-α mediated mechanism. Also, as Fxr has been observed to be decreased in NALFD [36], it is possible Fxr-dependent mechanisms regulate Slco expression. Fxr regulates mouse Slco1a1, 1a4 and 1a5 [37]. Pxr also regulates Slco1a4 expression in mice [38]. Similarly, human SLCO1B3 and 1A2 is regulated, in part, by FXR [39]. However, db/db mice did not demonstrate any significant differences in mRNA expression of Fxr and Pxr in liver, suggesting that in the observed Slco decrease in Db/Db mice may be due to Ppar-α activation, and not Pxr and Fxr alterations. These observed changes in Slco expression in db/db mice could be predicative of SLCO expression changes in livers of diabetic humans. Further studies, which reveal nuclear receptor binding to specific response elements present in Slco promoters, will further elucidate how these transporters are regulated in leptin/leptin receptor deficient diabetes models.

The regulation of renal transporter expression in mouse models of diabetes and obesity remains limited. Data in this manuscript and Cheng et al. [14] indicate that a severe diabetes phenotype alters renal transporter expression. It is intriguing that kidney transporter expression was substantially altered in this model, but minimal changes in renal pathology were observed. In humans SLC22A6 and SLC22A7 are predominant transporters localized to the basolateral membrane of renal proximal tubule cells [40]. The SLCs transport certain antibiotics like benzylpenicillin, antivirals and NSAIDs (Non-steroidal anti-inflammatory drugs). Slc22a7 expression was virtually undetectable in db/db male and female mice as compared to respective C57BKS controls, indicating the possibility of different renal elimination of substrates such as antibiotics, antivirals and non-steroidal anti-inflammatory drugs in this model. Slc22a6 and Slc22a2 expression was also downregulated in db/db mice, especially males. The mechanism for the observed Slc downregulation was not determined, however HNF1 has been described to regulate human and mouse SLC22A7/Slc22a7 and HNF4 has been described to regulate SLC22A7 in kidney [41,42].

Efflux transporters, in general, were upregulated in livers of db/db mice. Abcc3 transports mono-ionic bile acids such as glycocholate and taurocholate [43], as well as glucuronide or glutathione conjugates of certain drugs (e.g. APAP-G and morphine-3-glucuronide) [44]. Abcc3 and 4 expressions were significantly upregulated in db/db mice livers, in both genders. Abcc4 also transports bile acids, antiviral drugs, and cyclic nucleotides [15], but also contributes to the basolateral excretion of APAP-S [45,46]. Reisman et al. demonstrated increased plasma APAP-G and APAP-S concentrations correspond with increased Abcc3 and 4 protein expression, respectively [47]. Additionally, in a rat model of NASH, it was observed that increased Abcc3 expression enhanced urinary excretion of APAP-G [19]. Increased expression of Abcc3 and/or Abcc4 is associated with enhanced excretion of APAP metabolites [19,48]. In the present study, db/db mice had higher amounts of APAP-G and -S metabolites in urine, which was consistent with increased hepatic Abcc3 expression, and increased hepatic and renal Abcc4 expression. The reasons for higher excretion of APAP-G and APAP should be due to enhanced production of APAP-G and –S and/or enhanced basolateral excretion. Db/db mice also display increase in mRNA expression of the enzymes responsible for production of major conjugation metabolites like Ugt1a6 and Sult1a1 compared to C57BKS mice livers (Figure 8). Therefore, enhanced excretion of glucuronide and sulfate metabolites was expected. Overall, this data is consistent with published findings in children with NAFLD [22]. Increased APAP-G levels were observed in plasma and urine samples from children presenting with NAFLD [22]. Abcc1, 2, 4, and Abcg2 mRNA and/or protein expression was increased in liver, which is consistent with what was observed in livers of T2DM rats [49]. Abcc1 and Abcg2, along with Abcb1, can transport the antidiabetic drug rosiglitazone [50]. Severe liver injury has been reported in a person with T2DM [51] and cholestatic injury has also been observed after rosiglitazone therapy [52] – both suggesting hepatic clearance is necessary. Perhaps, differences in expression of these transporters in the diabetic liver could contribute to decreased hepatic clearance of rosiglitazone. An interesting observation is that rosiglitazone increases the incidence of cardiovascular disease in diabetic patients [53]. As its use is still approved, determining whether diabetes could impede rosiglitazone clearance is important for predicting persons at risk.

The transporters analyzed in this study are known to be regulated by different mechanisms, involving various transcription factors such as Ppar-α, Pxr, constitutive androstane receptor (Car), nuclear factor E2-related factor 2 (Nrf2), Fxr, and Hepatocyte nuclear factor 1-alpha (Hnf-1α). Li and Klaassen (2004) showed that HNF1α levels are critical for constitutive expression of Slco1b2 in mouse liver [54]. Also Slc22a6 and Slc22a7 expression in mouse kidneys is downregulated by targeted disruption HNF1α [55]. Significantly reduced expression of Slco1a1 in liver, along with Slc22a7 in kidney in db/db mice suggests that HNF1α levels or binding is decreased in these mice. Similarly, Abcc3 and Abcc4 efflux transporter expression is regulated in part by Nrf2-keap1 pathway in liver [24]. The present study clearly demonstrates that Abcc2-4 were upregulated in livers of db/db mice, which suggests activation of the Nrf2 and/or constitutive androstane pathways in these mice. Increased mRNA expression of Nrf2 and its target gene Gclc indicate that Nrf2-keap1 pathway is likely activated in db/db mice. The Nrf2-keap1 pathway is activated during periods of oxidative stress [56]. Also as reviewed by Rolo and Palmeira, diabetes is typically accompanied by increased production of free radicals, present findings suggests that oxidative stress may be present in diabetic liver [57]. Together, the data presented argue for additional future studies to better define nuclear receptor pathways that are upregulated in leptin/leptin receptor deficient models, which will aid in better understanding receptor-mediated mechanisms, which could regulate transporter expression in steatosis and T2DM. As reviewed by Klaassen and Slitt [38], Car and Pxr are also known for regulating Abcc2, 3, 5, 6 and Abcc2, 3 respectively. The observed increase in Abcc2, 3, 5, and 6 expression could be attributed to the observed increased in Car expression and activity, as shown in Figure 7.

Similar to the liver, transporter expression is markedly altered in kidneys of db/db mice. Maher and colleagues showed that targeted disruption in Hnf1α significantly downregulated Slc22a6, 7 and 8 and Slco1a1 mRNA in mice kidneys [55]. This indicates that db/db mice might have differential expression or binding of Hnf1α. Also, these mice have severe hyperglycemia. During normal course, almost all of the glucose is absorbed from the nephrons during urine formation. But due to overwhelming amounts of glucose in glomerular filtrate, kidneys are unable to absorb it and thus excrete glucose in urine. This hyperglycemic urine may cause some alterations in transporter expression in kidneys.

## Conclusions

Data illustrated in the present study illustrate a comprehensive, panoramic view of how a severe diabetes phenotype affects liver and kidney transporter expression in mice. These changes were associated with altered excretion of the APAP metabolites, APAP-G and –S, which is consistent with a recent publication in children with NAFLD [22]. Changes in transporter expression could, in part, explain why certain drugs have altered ADME in humans with diabetes. In summary, we demonstrate that db/db mice, which exhibit a severe diabetes phenotype display marked alterations in transporter expression in liver and kidney.

## Methods

### Animals and husbandry

Seven-week-old C57BKS and db/db (BKS.Cg-m +/+ Leprdb/J, Jax mice stock # 000642) mice (n = 8, for each strain and gender) were purchased from Jackson Laboratories (Bar Harbor, ME). Mice were housed for 2 weeks under a constant dark/light cycle (12 hr/12 hr) and given food and water *ad libitum.* The mice were fed the same feed (LabDiet 5 K20) as at Jackson laboratories in order to maintain a consistent food source. During acclimation, body weight and blood glucose levels (Glucose meter, Bayer Healthcare, Tarrytown, NY) were measured each week. After 2 weeks of acclimation mice were anesthetized by isofluorane inhalation – 9 weeks of age was selected to evaluate expression in db/db mice because the mice have reached maturity, and exhibit significantly elevated blood glucose levels along with hepatic steatosis, as well as, to compare previous transporter expression observations in ob/ob mice [14]. Blood was collected and serum was obtained after centrifugation at 2300xg for 5 minutes at 4°C. Livers and kidneys were collected, snap frozen in liquid nitrogen, and stored at −80°C for future analysis. Experiments were approved by The University of Rhode Island Institutional Animal Care and Use Committee (IACUC).

### RNA extraction

Total RNA from liver and kidney was isolated by phenol-chloroform extraction using RNA Bee reagent (Tel-Test Inc, Friendswood, TX) according to the manufacturer’s protocol. RNA concentration was quantified by absorbance at 260 nm using a spectrophotometer (Nanodrop ND1000, Thermo Fisher Scientific, Waltham, MA) and the samples were diluted to 1 μg/μL. Formaldehyde–agarose gel electrophoresis followed by UV illumination was used to visualize RNA and confirm integrity.

### Oligonucleotide probesets for branched DNA signal amplification (bDNA) assay

Probe sets for mouse Abcc1-6, Slc22a6, 7, 8, Slco1a1, 1a4, 1b2, 1a6, 2b1, Nrf2, Gclc, Fxr, Shp, Ppar-α, Car, Pxr, Cyp3a11, Cyp2b10 and Cyp4a14 have been described previously [23,33,58,59]. Oligonucleotide probesets required for the assay were graciously donated by Dr. Curtis Klaassen (University of Kansas Medical Center, Kansas City, KS).

### bDNA assay

The Branched DNA assay has been employed in multiple studies to evaluate relative biotransformation enzyme and transporter mRNA expression [19,23,33]. All reagents for analysis including lysis buffer, amplifier/label probe diluent and substrate solution were supplied in the QuantiGene 1.0 assay kit (Panomics, Fremont, CA). Oligonucleotides were first dissolved in 10 mM Tris–HCl (pH 8.0) with 1 mM EDTA and were diluted 1:100 in lysis buffer before use [60]. On day one, total RNA samples (10 μg, 1 μg/μL) were added to wells containing 50 μL of capture hybridization buffer and 50 μL of diluted probe set. The RNA was allowed to hybridize overnight with probe set at 53°C. On day two, subsequent hybridization steps were followed as mentioned in manufacturer’s protocol, and fluorescence was measured with a GloRunner^TM^ microplate luminometer interfaced with GloRunner DXL Software (Turner Biosystems, Sunnywale, CA). The fluorescence for each well was reported as relative light units (RLU) per 10 μg of total RNA.

### Preparation of crude membrane preparations from liver and kidneys

Crude membrane fractions were prepared from livers and kidney, as this fraction has been previously described for measurement of transporter expression [24,61]. Approximately 50 mg of tissue was homogenized in Sucrose-Tris (ST) buffer (250 mM sucrose 10 mM Tris–HCl buffer, pH 7.4) and containing protease inhibitor cocktail (2 μg/mL, Sigma-Aldrich, Co, St. Louis, MO). Homogenates were centrifuged at 100,000 g for 60 min at 4°C. ST buffer (200 μl) was used to re-suspend the resulting pellet. Protein concentration of the crude membrane fractions was determined using the Biorad DC protein assay reagent (Bio-Rad Laboratories, Hercules, CA).

### Western blot analysis of crude membrane fractions

Western blot analysis was used for identification and quantification of specific transport proteins. Crude membrane fractions (50 μg protein/well) were electrophoretically resolved by SDS-Polyacrylamide gel (4-20%) electrophoresis. Proteins were transblotted onto polyvinylidene fluoride (PVDF) membrane (Millipore, Bedford, MA) at 100 V for 45 minutes. The membrane was blocked overnight at 4°C with 2% non-fat dry milk in phosphate-buffered saline with 0.05% Tween 20 (PBS/T). The membrane was then incubated with primary antibody in PBS/T for 3 hrs at room temperature. Following three washes in PBS/T, the membrane was incubated with species-specific peroxidase-labeled secondary antibody diluted in PBS/T for 1 hour at room temperature. The specific information about the source, dilution, type, and molecular weight of primary and secondary antibodies is detailed in supplemental information (Additional file 2: Table S1). After incubation with secondary antibody, membranes were washed three times in PBS/T, incubated with ECL + fluorescence Reagent (GE Healthcare, Buckinghamshire, UK), and developed using autoradiography. Protein bands on autoradiographs were quantified using Quantity One^®^ software v4.6.3 (Biorad, Hercules, CA). B-actin or Gapdh were used as loading controls for western blotting.

### Immunohistochemical staining

Abcc3 expression and localization were evaluated because increased Abcc3 protein expression in liver is associated with changes in vectorial excretion of acetaminophen-glucuronide [25]. Frozen tissues were cut into 5 μm sections on a Vibratome cryostat and were fixed in 4% paraformaldehyde in phosphate-buffered saline (PBS) for 5 minutes. Sections were washed twice with PBS, for 5 minutes at room temperature, and then washed once with PBS containing 0.2% Triton X-100 (PBS-Triton) for 5 minutes. Next, sections were incubated with blocking agent (5% goat serum diluted in PBS-Triton) for 1 hour at room temperature. Blocking agent was removed and sections were then incubated with Abcc3 primary antibody (diluted 1:100 in blocking agent) for 2 hours at room temperature. Sections were washed thrice with PBS-Triton and then incubated with Alexafluor 488 goat anti-rat IgG antibodies diluted 1:100 in PBS-Triton and Rhodamine-conjugated phalloidin (Invitrogen Inc., Carlsbad, CA; diluted 1:200) for 1 hour at room temperature in dark. After incubation, sections were washed twice with PBS-Triton, followed by a wash with PBS, and then double-deionized water. Sections were allowed to air dry and were mounted with Prolong® Gold containing DAPI (Invitrogen Inc., Carlsbad, CA).

### Acetaminophen (APAP) disposition in C57BKS and db/db male mice

Ten week old C57BKS and db/db male mice (n = 5) were obtained from Jackson Laboratories (Bar Harbor, ME). Only male mice were used for this study, as both genders exhibited increased liver Abcc3 and 4 expressions, and APAP disposition studies in rodents are typically performed using males. After two weeks acclimation, mice were administered APAP (100 mg/kg, po) in 0.9% saline. Immediately after dosing, mice were housed individually in metabolic cages equipped with urine collection trays that kept cool with custom ice packs (Techniplast, USA). The total urine volume over 24 hrs was measured. To precipitate proteins in urine, samples (100 μl) were diluted with 200 μl cold methanol and centrifugated at 4,000 g for 30 min at 4°C. The resulting supernatants were collected (250 μl) and diluted with 500 μl mobile phase. After mixed, the samples were centrifuged at 4,000 g for 10 min at 4°C. 100 μl of the supernatant is used for HPLC analysis. The column used for HPLC analysis was Eclipse XDB-C18 (4.6 mm x 15 cm, 3.5 μm). The mobile phase A contained 8% methanol and 1% acetic acid in water, and B contained 50% methanol in water. For first 5 min, mobile phase B was maintained at 100% followed by linear gradient of 10 min, ending in 25% of mobile phase B.

### Statistical analysis

Statistically significant differences between groups were determined by one-way ANOVA followed by a Newman-Keuls post hoc test. Unless otherwise stated, all data is presented as mean ± SEM for n = eight mice per group. For APAP disposition data, t-test was used for statistical significance. Values with P≤0.05 were considered statistically significant.

## Abbreviations

Abc, ATP binding cassette; ADME, Absorption distribution metabolism and elimination; APAP, Acetaminophen; APAP-CG/CYS, Acetaminophen cysteinylglycine/cysteine; APAP-G, Acetaminophen glucuronide; APAP-NAC, Acetaminophen N-acetyl L-cysteine; APAP-S, Acetaminophen sulfate; Car, Constitutive androstane receptor; Cyp, Cytochrome P450; DAPI, 4’, 6-diamidino-2-phenylindole; DILI, Drug-induced liver injury; DME, Drug metabolizing enzyme; FXR, Farnesoid-X- receptor; Gclc, Glutamate cysteine ligase; HNF1α, Hepatocyte nuclear factor-1α; Icam-1, Intercellular adhesion molecule-1; IL, Interleukin; Mcp, Monocyte chemoattractant protein; Mdr, Multidrug resistance protein; NAFLD, Non-alcoholic fatty liver disease; NASH, Non-alcoholic steatohepatitis; PBS, Phosphate buffered saline; PBS/T, Phosphate buffered saline with Tween 20; Ppar, Peroxisome proliferator activated receptor; PXR, Pregnane-X-receptor; Shp, Small heterodimer partner; ST buffer, Sucrose tris buffer; T1DM, Type-1 diabetes mellitus, T2DM, type-2 diabetes mellitus; TG, Triglycerides; Tnf-α, Tumor necrosis factor-α.

## Competing interests

The authors declare that they have no competing interests.

## Authors' contributions

VRM performed all experiments with mRNA and protein expression and immunohistochemistry, and drafted the manuscript. XW analyzed urine samples for APAP and metabolites. PET developed method for APAP analysis by HPLC. ALS, LMA and VRM designed the experiment, and contributed to writing of manuscript. All authors read and approved the final manuscript.

## Supplementary Material

Additional file 1 **Figure S1.** Title of data: Moderate steatosis db/db mice. Description of data: Hematoxylin and eosin staining showing mild to moderate steatosis in female and male db/db mice as compared to C57BKS mice livers. Click here for file

Additional file 2 **Table S1.** Title of data: Primary antibodies for western blot. Description of data: Type, dilution, molecular weight and sources of primary antibodies for western blot. Click here for file
