# Supplementary figures and images for: Severe diabetes and leptin resistance cause differential hepatic and renal transporter expression in mice
Source: Comp Hepatol. 2012 Apr 23;11:1. doi: 10.1186/1476-5926-11-1 (PMC3416584; doi:10.1186/1476-5926-11-1)

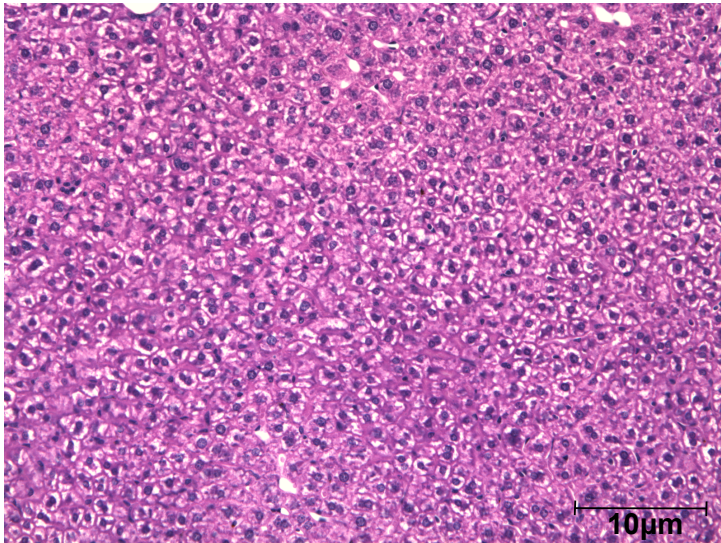

**C57BKS Female**

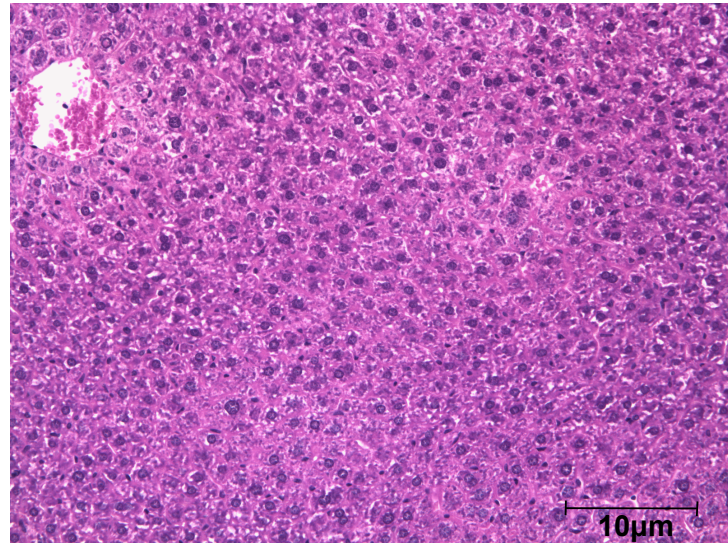

**C57BKS Male**

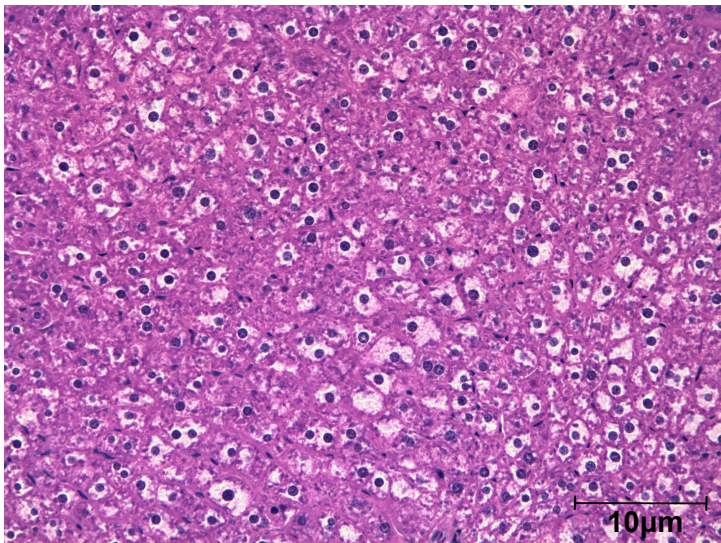

**Db/db Female**

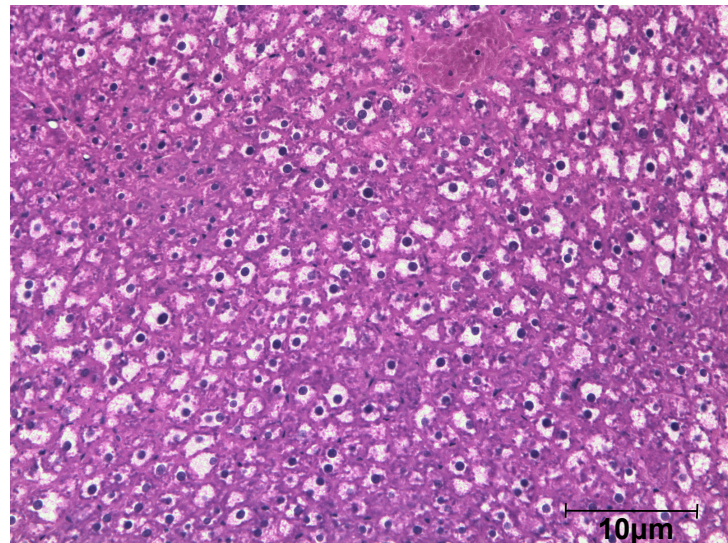

**Db/db Male**

Supplement: Additional file 1 — Figure S1. Title of data: Moderate steatosis db/db mice. Description of data: Hematoxylin and eosin staining showing mild to moderate steatosis in female and male db/db mice as compared to C57BKS mice livers. [file 1476-5926-11-1-S1.pdf]
