# Supplementary material for: Severe diabetes and leptin resistance cause differential hepatic and renal transporter expression in mice
Source: Comp Hepatol. 2012 Apr 23;11:1. doi: 10.1186/1476-5926-11-1 (PMC3416584; doi:10.1186/1476-5926-11-1)
Supplement: Additional file 2 — Table S1. Title of data: Primary antibodies for western blot. Description of data: Type, dilution, molecular weight and sources of primary antibodies for western blot. [file 1476-5926-11-1-S2.docx]

**Supplemental Table 1** Type, dilution, molecular weight, and sources of primary antibodies for western blots

| **Primary antibodies** | **Type** | **Dilution** | **Mol wt** | **Source** |
| --- | --- | --- | --- | --- |
| Slco1a1 |  | 1:1000 | ~70 | Dr. Curtis Klaassen, University of Kansas Medical Center |
| Slco1a4 |  | 1:1000 | ~70 | Abcam, MA |
| Slco1b2 |  | 1:2000 | ~70 | Dr. Curtis Klaassen, University of Kansas Medical Center |
| Abcc1 | MRPr1 | 1:2000 | ~190 | Dr. George Scheffer, VU Medical Center, Amsterdam |
| Abcc2 | M_2_III-5 | 1:600 | ~190 | Chemicon International- Millipore, MA |
| Abcc3 | M_3_II-2 | 1:2000 | ~180 | Dr. George Scheffer, VU Medical Center, Amsterdam |
| Abcc4 | M_4_I-10 | 1:2000 | ~160 | Dr. George Scheffer, VU Medical Center, Amsterdam |
| Abcc6 | M_6_II-68 | 1:1000 | ~165 | Dr. George Scheffer, VU Medical Center, Amsterdam |
| Abcg2 | BXP-53 | 1:2000 | ~75 | Dr. George Scheffer, VU Medical Center, Amsterdam |
| Gapdh/B-actin |  | 1:2000 | ~40 | Cell Signaling Technology, MA |
